# Localization and Function of the Cannabinoid CB1 Receptor in the Anterolateral Bed Nucleus of the Stria Terminalis

**DOI:** 10.1371/journal.pone.0008869

**Published:** 2010-01-25

**Authors:** Nagore Puente, Izaskun Elezgarai, Mathieu Lafourcade, Leire Reguero, Giovanni Marsicano, François Georges, Olivier J. Manzoni, Pedro Grandes

**Affiliations:** 1 Department of Neurosciences, Faculty of Medicine and Dentistry, Basque Country University, Bilbao, Spain; 2 INSERM U862 Equipe “Physiopathologie de la Transmission et de la Plasticité Synaptique”, Bordeaux, France; 3 “Endocannabinoids and Neuroadaptation”, INSERM U862 NeuroCentre Magendie, Université Bordeaux 2, Bordeaux, France; Vrije Universiteit Amsterdam, Netherlands

## Abstract

**Background:**

The bed nucleus of the stria terminalis (BNST) is involved in behaviors related to natural reward, drug addiction and stress. In spite of the emerging role of the endogenous cannabinoid (eCB) system in these behaviors, little is known about the anatomy and function of this system in the anterolateral BNST (alBNST). The aim of this study was to provide a detailed morphological characterization of the localization of the cannabinoid 1 (CB1) receptor a necessary step toward a better understanding of the physiological roles of the eCB system in this region of the brain.

**Methodology/Principal Findings:**

We have combined anatomical approaches at the confocal and electron microscopy level to *ex-vivo* electrophysiological techniques. Here, we report that CB1 is localized on presynaptic membranes of about 55% of immunopositive synaptic terminals for the vesicular glutamate transporter 1 (vGluT1), which contain abundant spherical, clear synaptic vesicles and make asymmetrical synapses with alBNST neurons. About 64% of vGluT1 immunonegative synaptic terminals show CB1 immunolabeling. Furthermore, 30% and 35% of presynaptic boutons localize CB1 in alBNST of conditional mutant mice lacking CB1 mainly from GABAergic neurons (GABA-CB1-KO mice) and mainly from cortical glutamatergic neurons (Glu-CB1-KO mice), respectively. Extracellular field recordings and whole cell patch clamp in the alBNST rat brain slice preparation revealed that activation of CB1 strongly inhibits excitatory and inhibitory synaptic transmission.

**Conclusions/Significance:**

This study supports the anterolateral BNST as a potential neuronal substrate of the effects of cannabinoids on stress-related behaviors.

## Introduction

The bed nucleus of the stria terminalis (BNST) is a key structure of the extended amygdala's network involved in behaviors related to natural reward, drug addiction and stress [1]–[7]. Based on cyto and chemoarchitectonic features, the BNST is composed of several neuronal nuclei organized in the anterior and posterior divisions with distinct connectivity and functional implications. Within the anterior division, we have focused in this study on the anterolateral area because of the broad connectivity with systems involved in stress signal processing. This area is innervated densely by the central nucleus of the amygdala and the amygdalar components of the main olfactory system (anterior amygdalar, anterior cortical and postpiriform transition areas, and the anterior basomedial and posterior basolateral nuclei). It also receives a massive projection from gustatory and visceral sensory areas of the insular region [8]. Projections from olfactory, gustatory, insular and basolateral, basomedial and posterior amygdalar regions use glutamate as neurotransmitter and, thus, they exert an excitatory influence on their neuronal targets in the anterolateral BNST (alBNST) [8]. On the other hand, the efferent projections of the alBNST to autonomic hubs of the hypothalamus and lower brainstem [8] are organized into somatomotor, central autonomic and neuroendocrine systems [9]–[12] playing a critical role in stress processing. The alBNST outputs are inhibitory and use the neurotransmitter GABA. Indeed, a high neuronal expression of mRNA coding for glutamic acid decarboxylase (GAD) has been detected in this region [13]. However, despite the absence of vesicular glutamate transporter in the alBNST, an excitatory pathway to the ventral tegmental area originating from neurons placed in the anteromedial and anteroventral BNST regions has been reported [14], [15].

The endocannabinoid (eCB) system is a versatile modulatory system expressed widely throughout the central nervous system (CNS) and as such is involved in numerous fundamental physiological processes [16], [17]. Multiple recent evidence point toward a role for the eCB system in the behavioral responses to reward and stress. The implication of this system in reward is well documented and pharmacological, behavioral and genetic approaches all indicate its instrumental role in both acute and prolonged effects of drugs of abuse [18]. Furthermore, the anatomy and function of CB1 receptors in the BNST linked to the reward pathway are now beginning to be elucidated [15], [19]. Activation of CB1 receptors localized on excitatory presynaptic boutons of prefrontal infralimbic cortical neurons making synapses with BNST excitatory projecting neurons to VTA, inhibits VTA dopamine neurons upon infralimbic cortical stimulation, suggesting a new neuronal circuitry for the actions of cannabinoids [14], [15].

As to stress-related behaviors, the eCB system negatively modulates the stress-induced activation of hypothalamic-pituitary-adrenal axis [20]. Moreover, inhibition of the degradation or the reuptake of the two major endocannabinoids (2-arachidonoyl-glycerol and anandamide) reduces anxiety and depression-like behaviors in rodent models [21]–[23].

The presence of CB1 mRNA in projecting neurons to the anterolateral BNST as well as in intrinsic inhibitory neurons of this region [24] opens the question about the anatomy and functional role of the eCB system in the processing information of stress signals in the anterolateral BNST. In the present study we combined confocal microscopy, electron microscopy and *ex vivo* electrophysiological recordings to investigate the role of CB1 receptors in the rat anterolateral BNST. We found CB1 receptors localized on presynaptic membranes of excitatory and inhibitory-like synaptic terminals. Moreover, activation of these receptors by exogenous agonists strongly inhibited evoked excitatory and inhibitory post-synaptic currents. Together, the involvement of presynaptic CB1 in the modulation of excitatory and inhibitory systems converging onto alBNST neurons projecting to hypothalamic nuclei [25] may be crucial in the regulation of stress responses.

## Results

### Immunolocalization of CB1 in the Anterolateral BNST

**Figure 1:**
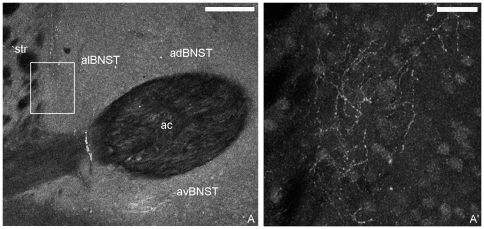
Confocal CB1 immunofluorescence in the anterior division of the rat BNST. In A, intense CB1 labeling was in the anterodorsal (adBNST), anteroventral (avBNST) and anterolateral (alBNST) BNST around the anterior commissure (ac). In A′, an enlargement of the area framed in A shows CB1 immunoreactive varicose fibers in alBNST. Str: striatum. Scale bars: A: 200 µm; A′: 30 µm.

Confocal and electron microscopy approaches were used to define the localization of CB1 in the alBNST. CB1 immunofluorescence was observed throughout the anterior division of BNST, particularly, in the medial (anteroventral, anterodorsal areas) and lateral groups (anterolateral area) (Fig. 1A). At higher magnification, dotty elements and varicose fibers with beaded protrusions formed a CB1 immunoreactive mesh-like in the neuropil of the anterolateral area close to the dorsal striatum and adjacent to the internal capsule (Fig. 1A, A′).

**Figure 2:**
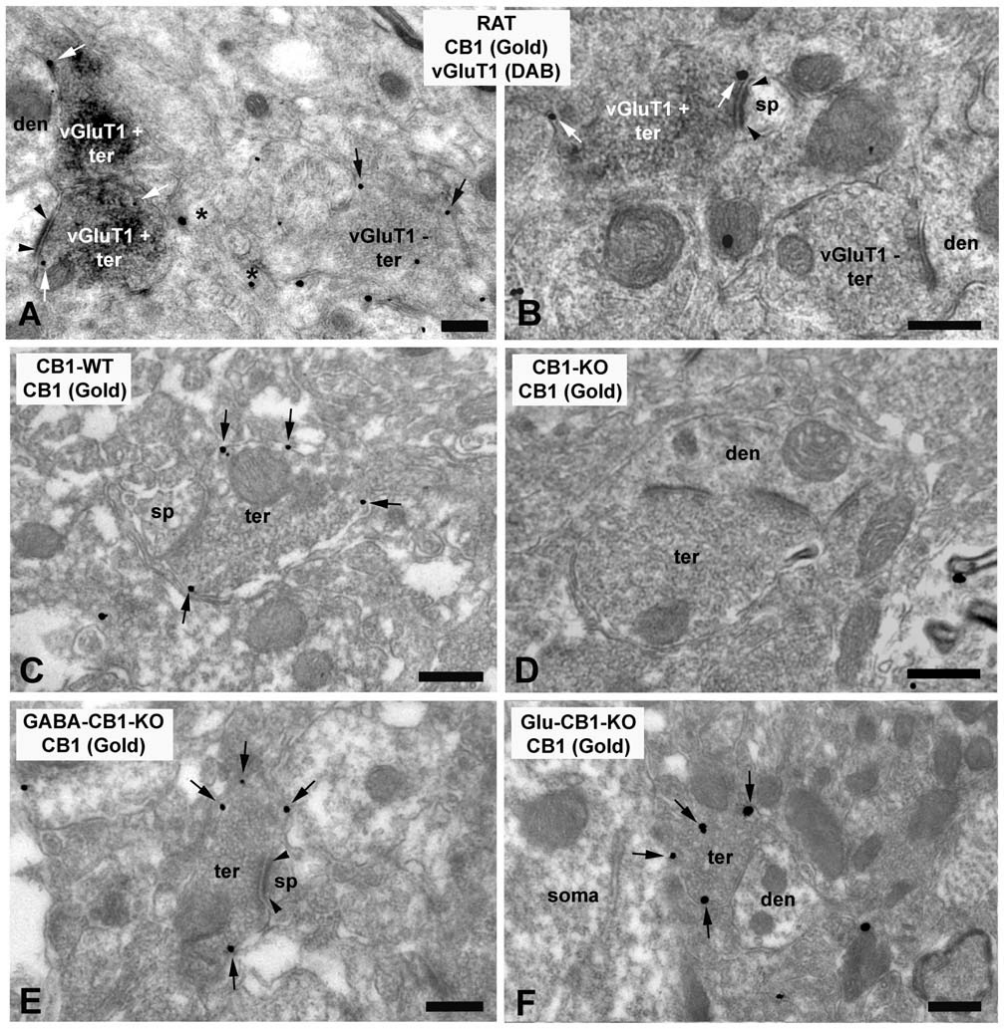
Immunolocalization of CB1 and vGluT1 in the rat anterolateral BNST (A, B). Double labeling by combining a preembedding immunogold (CB1) and an immunoperoxidase (vGluT1) method for electron microscopy. vGluT1 immunoreactive presynaptic axonal terminals (vGluT1 + ter) making synapses with small dendrites (den) and dendritic spines (sp) localized CB1 immunoparticles at their perisynaptic and extrasynaptic membranes (white arrows). Observe in A, CB1 immunolabeling (black arrows) in a vGluT1 immunonegative synaptic bouton (vGluT1–ter); and in B, a vGluT1–synaptic terminal (vGluT1–ter) CB1 immunonegative. Small unmyelinated axons also showed CB1 immunoparticles (asterisks in A). The CB1 immunolabeled (arrows) synaptic terminals (ter) making synapses with small dendrites (den) and dendritic spines (sp) observed in wild-type mice (C) disappeared in CB1 −/− BNST tissue (D). CB1 immunolabeling (arrows) was detected in presynaptic boutons (ter) forming excitatory asymmetrical synapses with dendritic spines (sp) in the anterolateral BNST of GABA-CB1-KO mutant mice (E). Similarly, CB1 (arrows) was revealed in inhibitory-like axonal terminals (ter) making symmetrical synapses with small dendrites (den) in the anterolateral BNST of Glu-CB1-KO mutant mice (F). Scale bars: 0.4 µm.

**Figure 3:**
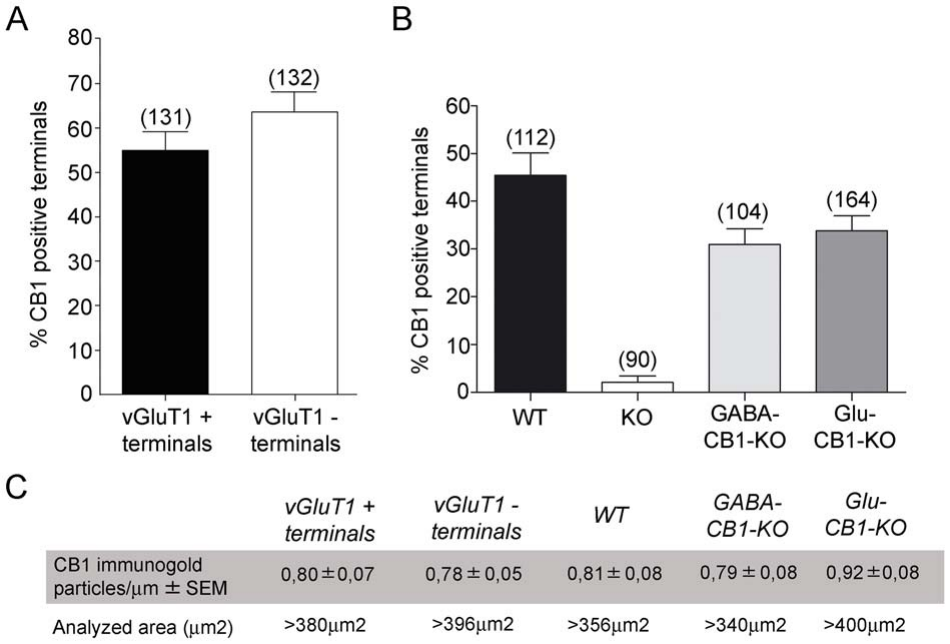
A, Percentages of vGluT1 + and vGluT1 − synaptic terminals localizing CB1 in the rat anterolateral BNST. 54.52% of the vGluT1 + and 63.55% of the vGluT1 − presynaptic boutons localized CB1 silver-intensified gold particles along their membranes. B, Percentages of CB1 immunopositive synaptic terminals in the anterolateral BNST of wild-type (45.40±4.737%), CB1-KO (2.063±1.411%), GABA-CB1-KO (30.93±3.309%) and Glu-CB1-KO (33.83±3.099) mutant mice. Immunolabeling was negligible in CB1-KO tissue. C, Density of CB1 immunoparticles in vGluT1 + and vGluT1 − synaptic membranes after subtraction of background labeling (0.08 particles/µm), and area analyzed in each tissue condition.

Double preembedding immunoelectron microscopy was used for the study of the precise localization of CB1 and vGluT1 in the anterolateral BNST (Fig. 2A, C). About 55% of the presynaptic boutons immunoreactive for vGluT1 had CB1 silver-intensified gold particles along their membranes (Figure 3). They contained round and clear synaptic vesicles and made excitatory asymmetrical synapses with postsynaptic dendritic spines and small dendrites (Fig. 2A, C). Approximately 64% of immunonegative vGluT1 synaptic terminals showed CB1 immunolabeling (Figure 3; Fig. 2A). The density of silver-intensified gold particles in immunopositive and immunonegative vGluT1 synaptic terminals was 0.80 particles/µm and 0.79 particles/µm, respectively, being this difference not statistically significant (p>0.05). CB1 immunoparticles were consistently associated with portions of presynaptic membranes away from the active zones. CB1 receptors were also localized in membranes of cross-sectioned thin unmyelinated axons (Fig. 2A).

Although *in situ* hybridization studies have shown that the vGluT1 is the predominant vesicular glutamate transporter in brain regions of cortical origin projecting to the alBNST, vGluT2 and vGluT3 mRNAs are also present in the basomedial amygdala and other cortical regions sending axons to the alBNST [29]. Based on this, we evaluated to what extent the presence of CB1 in vGluT1 + and vGluT1 - synaptic profiles correlates with the localization of CB1 in excitatory and inhibitory synaptic terminals in the alBNST. For this purpose, we used conditional mutant mice lacking CB1 mainly from cortical glutamatergic neurons (Glu-CB1-KO mice), and mainly from GABAergic neurons (GABA-CB1-KO mice) [27], [28]. First, we analyzed the subcellular CB1 distribution in alBNST of wild type mice. The pattern was identical to the rat, with CB1 immunoparticles on presynaptic boutons making asymmetrical and symmetrical synapses with small dendrites or dendritic spines (Fig. 2C). CB1 immunoreactivity was not observed in alBNST synapses of CB1 deficient mice (Fig. 2D). In the conditional GABA-CB1-KO mutants, about 30% of the presynaptic terminals localized CB1 immunoparticles (Figure 3) and formed synaptic contacts showing typical ultrastructural features of excitatory asymmetrical synapses (Fig. 2E). As to the Glu-CB1-KO mice, CB1 was in approximately 35% of the synaptic boutons (Figure 3) that were characterized by their inhibitory-like symmetrical synapses (Fig. 2F).

No specific staining was detected in sections in which the primary goat anti-CB1 antibodies were replaced by goat serum (0.069 particles/µm) (not shown).

### Cannabinoids Acting at Presynaptic CB1 Inhibit Glutamatergic Transmission in the Anterolateral BNST Slice Preparation

The CB1 immunolabeling at vGluT1 + axon terminals forming asymmetrical synapses and our *in vivo* observations [15] strongly suggest the presence of presynaptic CB1 on excitatory afferents impinging on BNST neurons. We directly tested this possibility using the slice preparation of the BNST. Both whole cell voltage clamp and extracellular field recordings were performed in the alBNST-containing slice preparations.

**Figure 4:**
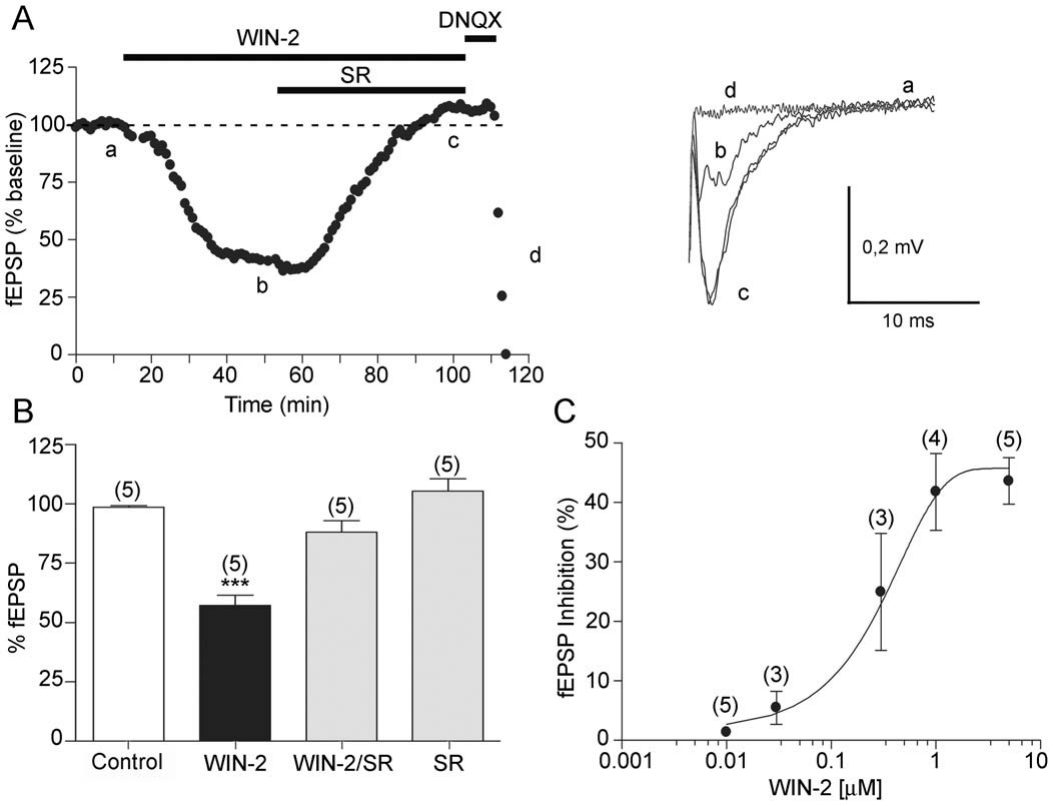
Pharmacological characterization of the CB1 mediated inhibition in the rat anterolateral BNST. A, *Left;* typical experiment (time course) where the CB1 agonist, WIN-2 (5 µM) reduces the fEPSP and the selective CB1 antagonist SR141716A (SR, 4 µM) reverses the WIN-2 induced inhibition. The non-NMDA ionotropic glutamate receptor antagonist DNQX (20 µM) eliminated the fEPSP confirming its synaptic origin. *Right;* superimposed traces (average of 20 consecutive fEPSPs taken at the time indicated on the time course) showing the inhibition caused by WIN-2 (b) and its reversion by SR (c). Calibration bars: x: 10 ms, y: 0.2 mV. B, summary of all the experiments performed with WIN-2 and SR: The fEPSP was 98.64±0.6590% of baseline in control (n = 5), 57.21±4.335% (n = 5; p = 0.0008; ***: p<0.05) with WIN-2 at 60 min, 88.09±4.83% (n = 5; p = 0.0727, ns: p>0.05) with WIN-2/SR at 120 min, and 105.4±5.288% (n = 5; p = 0.2246; ns: p>0.05) 30 min after perfusing only with SR. C, Dose response curve measured 20 min after beginning WIN-2 application. Each point is expressed as the percentage of inhibition of its basal value. The EC_50_ for WIN-2 was 251.18+/−9.50 nM.

First, extracellular field recordings were performed to measure the effects of a CB receptor agonist WIN55,212,2 (WIN-2) on field excitatory postsynaptic potentials (fEPSP) evoked in the presence of the GABA-A antagonist picrotoxin (100 µM), by stimulating the internal capsule [19], [25]. WIN-2 (5 µM) markedly inhibited the evoked fEPSP as shown in Fig. 4A. This depression was completely reversed by the selective CB1 antagonist SR141716A (Fig. 4A, B; 4 µM) [30], showing the implication of cannabinoid receptors of the CB1 subtype. No significant effect was observed when SR141716A was administered alone (Fig. 4B, n = 5 paired t test p = 0.2246; ns: p>0.05). We performed full dose-response curves of the effects of the WIN-2. The CB receptor agonist inhibited fEPSP in a dose-dependent manner with an EC_50_ of 251±9 nM (Fig. 4C). Thus, the inhibitory effects of WIN-2 can be attributed to the activation of CB1 receptors in accord with previous reports [31]–[33]. As shown in Fig. 4A, the glutamatergic nature of the extracellular fEPSP was confirmed at the end of each experiments through the application of the non-NMDA ionotropic glutamate receptor antagonist DNQX (20 µM), which completely blocked the synaptic “N2 component” without altering the non-synaptic “N1 component” [25].

**Figure 5:**
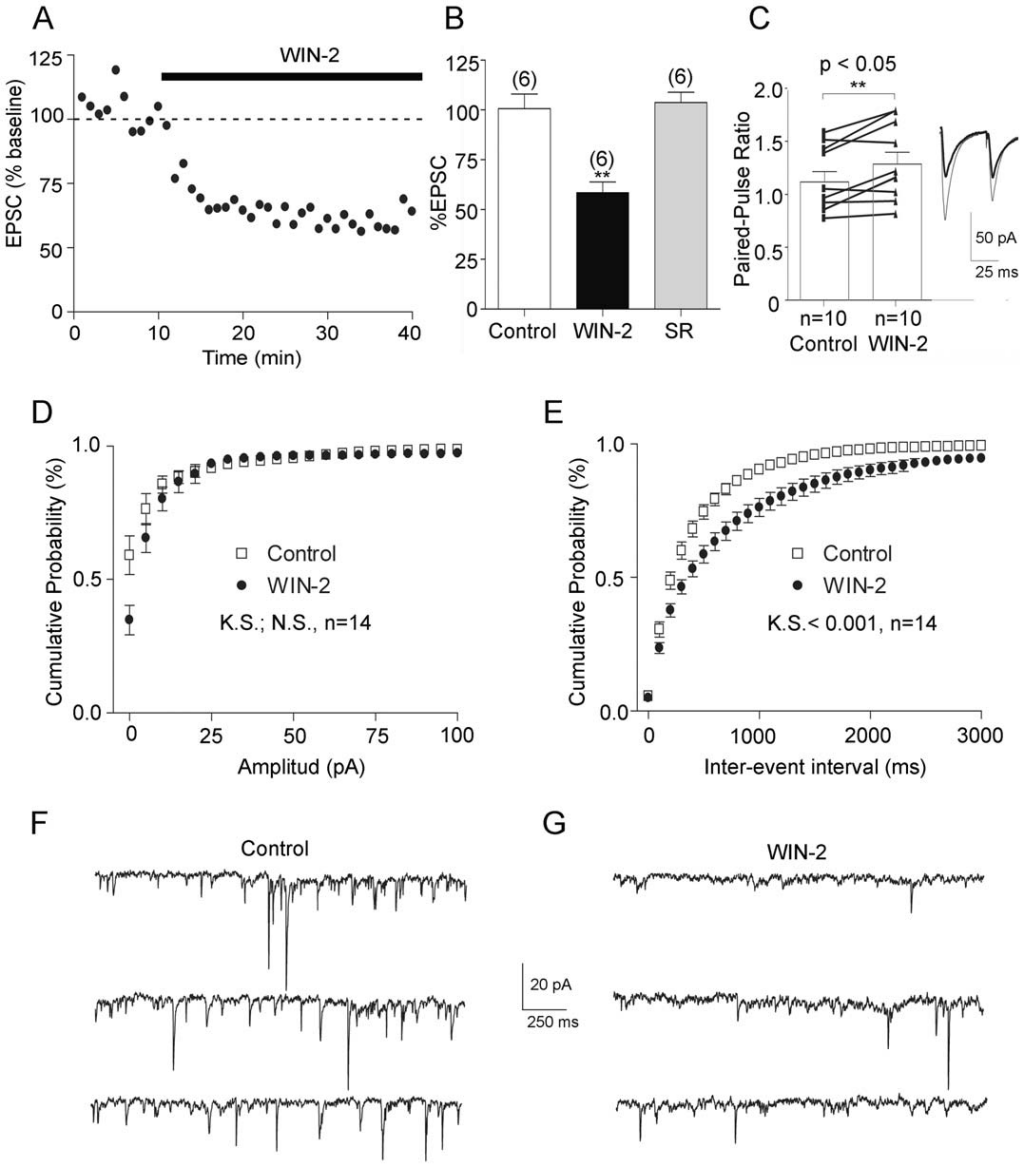
CB1 inhibits glutamatergic synapses in the anterolateral BNST at a presynaptic locus. A, time course of 1 µM WIN-2-induced inhibition of evoked EPSC normalized to baseline. B, Summary of all experiments performed as described in A: EPSC was 100.6±7.355% (n = 6) of baseline in control, 58.46±5.406% (n = 6; p = 0.03 t test *: p<0.05) at 60 min after WIN-2 and 103.7±5.230% (n = 6; p = 0.8483; ns: p>0.05) at 120 min after co-perfusion with SR C *left*, paired-pulse ratio were augmented during WIN-2 perfusion (n = 10; paired t test p = 0.0065; **: p<0.05). PPR was calculated with 60 sweeps i.e. 10 min before and 20 min after WIN-2.; *right*, representative EPSC traces from an alBNST neuron before and after WIN-2 application, average of 30 consecutive traces. Observe that the inhibition of the first EPSC by WIN-2 is larger than the second one. Calibration bars: x: 25 ms, y: 50 pA. D, bath perfusion of WIN-2 did not change the distribution of sEPSCs amplitude (n = 14). E, the distribution of the time intervals between successive sEPSCs in these neurons revealed that the sEPSCs frequency was reduced during WIN-2 application. (Kolmogorov-Smirnov test p<0.0001). Representative consecutive 2-sec current sweeps from a cell where sEPSCs were recorded in control (F) and 30 min after the start of the WIN-2 application (G). Calibration bars: x: 250 ms, y: 20 pA.

Whole cell voltage clamp recordings (CH_3_O_3_SCs-based intracellular medium, holding potential −70 mV) yielded similar results. Indeed, bath perfusion of WIN-2 (1 µM) inhibited evoked excitatory postsynaptic currents (EPSCs) as shown in Fig. 5A. This suppression (Fig. 5B, n = 6, paired t test p = 0.03; *: p<0.05) was also reversed by SR141716A (Fig. 5B, n = 6, paired t test p = 0.8483; ns: p>0.05).

To functionally assess the origin of the CB1-mediated depression, the variation of the paired-pulse ratio (PPR) of excitatory transmission, a presynaptic phenomenon thought to reflect changes in transmitter release and sensitive to presynaptic manipulations, was measured during bath application of the CB1 agonist. In agreement with a presynaptic site of action for CB1, the depression of evoked release induced by the CB receptor agonist was accompanied by an increase of the PPR (Fig. 5C, n = 10, paired t test p = 0.0067; **: p<0.05). Further confirming the presynaptic origin of the CB1-mediated inhibition, we observed that the frequency but not the amplitude of spontaneous EPSCs was reduced following bath perfusion of WIN-2 (Fig. 5D–G, n = 14, Kolmogorov-Smirnov test p<0.0001).

### CB1-Mediated Depression at Inhibitory Synapses

**Figure 6:**
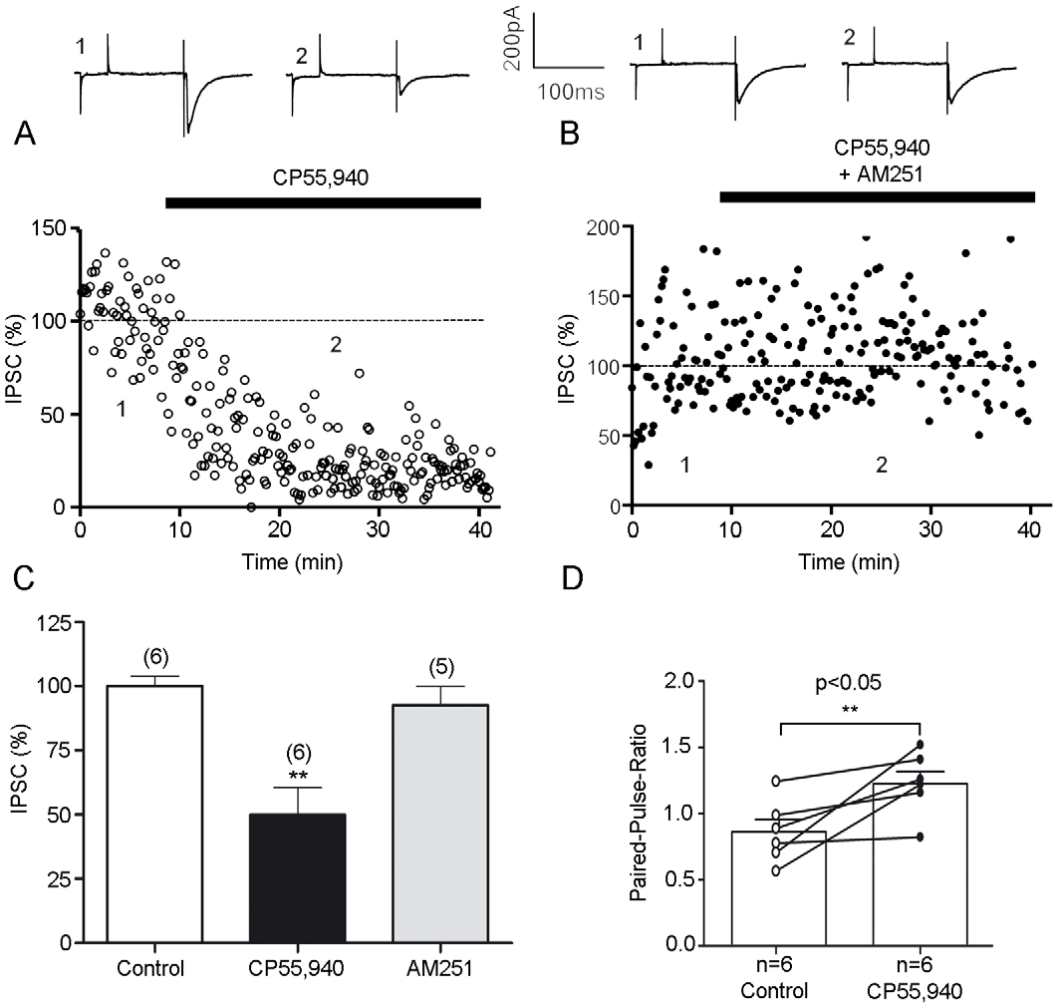
Activation of presynaptic cannabinoid receptors diminishes GABA_A_ IPSCs recorded in anterolateral BNST neurons. A, Typical experiment (time course) where the CB1 agonist, CP55,940 (10 µM) inhibited IPSCs. Above traces represent the average of 10 consecutive EPSCs taken at the times indicated on the time-course graph. B, The inhibitory effects of CP55,940 on evoked IPSCs were blocked by pre-treatment with the selective CB1 antagonist AM251 (4 µM) in agreement with the involvement of CB1. Above traces represent the average of 10 consecutive IPSCs taken at the times indicated on the time-course graph. All experiments were conducted in the presence of DNQX 20 µM and APV 50 µm. C, Summary of all experiments performed as described in A and B: IPSC was 100.1±3.647% (n = 6) of baseline in control, 49.93±10.67% (n = 6; p = 0.0012 t test **: p<0.05) at 60 min after CP55,940 and 92.71±7.119% (n = 5; p = 0.3549; ns: p>0.05) at 120 min after perfusion with AM251. D, Paired-pulse ratio were augmented during CP55,940 perfusion (n = 6; paired t test p = 0.0312; *: p<0.05). PPR was calculated with 60 sweeps i.e. 10 min before and 20 min after CP55,940.

Since the anatomical evidence indicate the presence of CB1 in immunonegative vGluT1 as well as in Glu-CB1-KO synaptic terminals showing mostly ultrastructural features of inhibitory-like synapses in the alBNST, we next examined the role of CB1 in the modulation of the inhibitory synaptic transmission. Afferent stimulation in the presence of ionotropic glutamate receptor antagonists elicited IPSCs that were strongly inhibited by bath perfusion of the CB agonist CP55,940 (Fig. 6A, C; 10 µM). Between 10 and 20 minutes after CP55,940 evoked IPSCs were reduced on average to 49.93%+/−10.67% (Fig. 5C, n = 6, paired t test p = 0.0012; **: p<0.05) of basal value. The suppression effect of the CB agonist was totally prevented by co-perfusion with the selective CB1 antagonists 1-(2,4-dichlorophenyl)-5-(4-iodophenyl)-4-methyl-N-1-piperidinyl-1H-pyrazide-3-carboxamide (AM251; 4 µM, Fig. 5B, C, n = 5, paired t test p = 0.3549; ns: p>0.05) demonstrating the implication of cannabinoid receptors of the CB1 subtype. Thus, the inhibitory synaptic transmission in the anterolateral BNST is also modulated by CB1 activation.

We compared the paired-pulse ratio (PPR) in order to determine whether the CP55,940-mediated inhibition was presynaptic or postsynaptic in the inhibitory synapses. CP55,940 modified PPR of evoked IPSCs (Fig. 5D, n = 6; paired t test p = 0.0312; *: p<0.05).) indicating a presynaptic localization of cannabinoid CB1 receptors.

## Discussion

### CB1 Receptors Are Localized in Presynaptic Boutons in the Anterolateral BNST

The present study reports several findings regarding the ultrastructural localization and function of CB1 receptors in the anterolateral BNST, a division of the extended amygdala particularly involved in stress information processing. We found that CB1 receptors localize in vGluT1 + and vGluT1–synaptic terminals, suggesting the distribution of the receptor in excitatory and also in inhibitory-like boutons impinging on alBNST neurons. Indeed, previous studies have demonstrated that the alBNST receives a major excitatory projection from the basolateral amygdala [11] containing high hybridization signals for CB1 mRNA [24], [34], as well as from the insular cortical neurons [11] expressing low to moderate levels of CB1 mRNA [24]. The recent anatomical and functional identification of CB1 in excitatory synaptic terminals from infralimbic cortical neurons restricted to the anteromedial and anteroventral regions of the BNST [15], did not overlap with the CB1 synaptic terminal field herein described in the anterolateral BNST, thus likely serving different functions as we will discuss later. The anterolateral BNST also receives a GABAergic projection from the anterior amygdalar and central amygdaloid nuclei expressing low to high intensities of CB1 mRNA signals [24]. Also, *in situ* hybridization and immunocytochemistry have demonstrated CB1 in GABAergic BNST neurons [24], [35] which give off local axon collaterals within BNST [13]. All these pieces of evidence point to a plausible subcellular localization of CB1 at inhibitory presynatic terminals in the alBNST as described in other brain regions [36], [37]. Indeed, we have estimated in this study that about 64% of the vGluT1 immunonegative BNST synaptic terminals are CB1 immunoreactive. Also, we have preliminary data (not shown) of a co-localization of CB1 and green fluorescent protein (GFP) in alBNST of *knock-in* mice expressing GFP under the control of the glutamate decarboxylase 67 (GAD67) promoter [38].

The presence or absence of vGluT1 in synaptic terminals is not strictly correlated with the excitatory or inhibitory nature of the pathway. Methodologically, the possibility exits that the vGluT1 antibody gives some weak random background labeling resulting in false positive terminals, or, alternatively, that many of the very weakly labeled terminals may had been identified as false-negative in adjacent sections. Also, in addition to vGluT1, vGluT2 and vGluT3 mRNAs are expressed in cortical regions projecting to the alBNST [29]. These concerns prompted us to use conditional mutant mice lacking CB1 mainly from cortical glutamatergic neurons (Glu-CB1-KO mice), and mainly from GABAergic neurons (GABA-CB1-KO mice) [27], [28]. In these mutants, CB1 can be distinguished in synaptic terminals fulfilling ultrastructural features of inhibitory nature (Glu-CB1-KO mice) and excitatory nature (GABA-CB1-KO mice). However, the cortical-linked structures such as the amygdalar components of the main olfactory system and insular region [8] which do not express CB1 in the Glu-CB1-KO condition, are not the only excitatory pathways to alBNST. Indeed, vGluT2 containing-synaptic terminals from the thalamus and hypothalamus have been demonstrated impinging on the alBNST [29]. Therefore, it is plausible that the vGluT1–and Glu-CB1-KO inhibitory-like synaptic terminals localizing CB1 are overestimated due to the presence of CB1 in excitatory synaptic terminals of non-cortical origin.

### Cannabinoids-Induced Effects on Synaptic Transmission Are Controlled by Presynaptic CB1 Receptors in the Anterolateral BNST

We have shown *ex-vivo* that CB1 activation suppresses both excitatory and inhibitory synaptic transmission in the alBNST. CB1 receptor agonists, WIN-2 or CP55,940 depressed EPSC and IPSCs evoked by afferent stimulation. These suppression effects were strongly reduced by CB1 antagonists. When SR141716A and AM251 were administered alone, there was no significant effect observed indicating that endogenous cannabinoid system in the alBNST is not tonically active. Taken together, our results indicate a broad functional enrolment of CB1 receptors distributed in both excitatory and inhibitory synaptic terminals in the alBNST.

We found that cannabinoids increase the PPR of EPSCs and IPSCs, consistent with a decrease in release probability. In agreement with this idea, we further found that a CB receptor agonist decreases the frequency, but not the amplitude, of sEPSCs. The localization of CB1 in GABAergic and glutamatergic presynaptic terminals is well documented [36], [38]–[43], and many studies in different brain regions have demonstrated that cannabinoids acting at presynaptic CB1 receptors depress inhibitory and excitatory synaptic transmission [16], [44]–[47]. But beyond this, the reported observations herein settle recent evidence from our laboratories that the activation of CB1 receptors at excitatory synapses by 2-AG and anandamide generated by separate signals mediates two distinct forms of short-term (depolarization-supression of excitation) and long-term (long-term depression) synaptic plasticity in a single anterolateral BNST neuron [48]. Physiologically, the identification of this eCB-dependent synaptic plasticity in the anterolateral BNST could be regarded as a potential neuronal substrate of the effects of cannabinoids on stress-related behaviors [48]. The contribution of the presynaptic CB1-mediated modulation of the inhibitory synaptic transmission to the functional role of the anterolateral BNST remains to be elucidated.

### Behavioral Significance of Cannabinoids Action in the Anterolateral BNST

The BNST plays a key role in integrating information from stress input pathways and in turn regulating stress output and reward pathways. It has been suggested that the BNST acts as an intermediary for regions of the brain involved in perceiving stress and regions of the brain producing responses to stress [1], [9]. The ventral subiculum and cortical areas that transmit “processive” stressor information and the noradrenergic projections from the nucleus of the solitary tract that transmit “systemic” stressor information project to the paraventricular nucleus (PVN) via a relay in the BNST, as well as via direct projections to the hypothalamus. Actually, the anterolateral BNST projects to somatomotor (nucleus accumbens, substantia innominata, ventral tegmental area, retrorubral area and adjacent midbrain reticular nucleus), central autonomic (central amygdalar nucleus, dorsal lateral hypothalamic area, ventrolateral periaqueductal gray, parabrachial nucleus and nucleus of the solitary tract) and neuroendocrine (paraventricular and supraoptic nuclei, hypothalamic visceromotor pattern generator network) systems [10]–[12], [14].

The interactions between the BNST, PVN and ventral tegmental area (VTA) are complex, but one prediction would be that activation of the BNST by processive stressors (i.e., ventral subiculum and cortical areas) would result in increased output from BNST to the nucleus accumbens, VTA, PVN and/or the hypothalamic area surrounding the PVN. However, it is as yet unclear whether this would have a net excitatory or inhibitory effect on these regions. For example, while the majority of the BNST neurons are GABAergic, the demonstration of an excitatory projection from the anteroventral BNST to the VTA [14] could result in an increase of dopamine release in the nucleus accumbens. Thus it is plausible that BNST neurons play a role in the integration of information leading to adaptive changes in synaptic responses after administration of drugs of abuse. Indeed, this brain region plays a critical role in footshock-induced reinstatement of drug seeking [49]–[51], as well as morphine withdrawal-induced conditioned place aversion [52]. On the other hand, based on animal-wide genetic and pharmacological manipulations, cannabinoid signaling in the CNS acting through CB1 is thought to be actively involved also in the dopaminergic mesolimbic brain reward circuit. Pharmacological [53], [54] as well as genetic disruption [55], [56] of CB1 receptor activity elicits anxiety-like behaviors in rodents, suggestive of the existence of an intrinsic anxiolytic tone mediated by endogenous cannabinoids.

Our findings may serve as a basis for the interpretation of the mechanism by which cannabinoid receptors can regulate behavioral anxiety and stress-drug interactions in the BNST.

## Materials and Methods

### Animal Treatment

The protocols for animal care and use were approved by the appropriate Committee at the Basque Country University. Furthermore, the animal experimental procedures were carried out in accordance with the European Communities Council Directive of 22 July 2003 (2003/65/CE) and current Spanish regulations (Real Decreto 1201/2005, BOE 21-10-2005). Great efforts were made in order to minimize the number and suffering of the animals used.

### Confocal Immunofluorescence

Rats (n = 3, Sprague-Dawley 250 g) were deeply anesthetized with chloral hydrate (400 mg/kg body weight) and were transcardially perfused at room temperature (RT, 20–25°C) with phosphate-buffered saline (PBS, 0.1 M, pH 7.4) for 20 seconds, followed by 500 ml of 4% formaldehyde (freshly depolymerized from paraformaldehyde) and 0.2% picric acid in 0.1 M phosphate buffer (PB), pH 7.4, for 10–15 minutes. Then, brains were removed from the skull and postfixed in 4% formaldehyde for up to 1 hour at RT. 50 µm-thick coronal sections cut from alBNST in a vibratome, were washed and blocked in 0.1 M PBS containing 3% newborn calf serum (NCS), 0.5% Triton X-100 and 0.025% sodium azide for 1 hour at RT. We used polyclonal goat anti-CB1 antibodies (3 µg/ml in 3% NCS/PBS for 2 days at 4°C; CB1-Go-Af450-1; Frontier Science Co. Ltd; 1-777-12, Shinko-nishi, Ishikari, Hokkaido, Japan). Slices were then washed in blocking solution (3% NCS/PBS) and incubated with donkey anti-goat Alexa Fluor-488 (Molecular Probes, Eugene, OR, USA) at a working dilution of 1∶650 in 3% NCS/PBS for 1 day at 4°C. Slices were washed again in 0.1 M PBS and then mounted in Vectashield medium (Vector Laboratories, Burlingame, CA, USA), coverslipped, and imaged on a laser-scanning confocal microscope (Olympus Fluoview FV500). Photomicrographs were taken and presented using Adobe Photoshop (CS, Adobe Systems, San Jose, CA, USA).

### CB1 Immunocytochemistry for Electron Microscopy

Rats (n = 5, Sprague-Dawley 250 g) were deeply anesthetized as described above. In addition, 12 wild-type, Glu-CB1-KO, GABA-CB1-KO and CB1-KO mice (3 of each condition; [26]–[28]) were deeply anesthetized by i.p. injection of a mixture of Nembutal (5 mg/100 g body weight; Abbott Laboratories Inc., IL, USA) and urethane (130 mg/100 g body weight; Sigma-Aldrich, St. Louis, MO, USA). All animals were transcardially perfused with PBS (0.1 M, pH 7.4) and then fixed by 500 ml of 0.1% glutaraldehyde, 4% formaldehyde and 0.2% picric acid in PBS. Perfusates were used at 4°C. Tissue blocks were extensively rinsed in 0.1 M PBS (pH 7.4).

Coronal alBNST vibrosections were cut at 50 µm and collected in 0.1 M PBS (pH 7.4) at RT. Sections were preincubated in a blocking solution of 10% bovine serum albumin (BSA), 0.1% sodium azide and 0.02% saponin prepared in Tris-HCl buffered saline (TBS, pH 7.4) for 30 minutes at RT. A preembedding silver-intensified immunogold method and an immunoperoxidase method were used for the co-localization of CB1 and the vesicular glutamate transporter 1 (vGluT1) in rat alBNST sections. They were incubated in primary polyclonal goat anti-CB1 (3 µg/ml) and guinea pig anti-vGluT1 (1∶1,000; Millipore Bioscience Research Reagents, formerly Chemicon, Temecula, CA, USA) antibodies both in 10% BSA/TBS containing 0.1% sodium azide and 0.004% saponin on a shaker for 1 day at RT.

After several washes in 1% BSA/TBS, tissue sections were incubated in a secondary 1.4 nm gold-labeled rabbit anti-goat IgG (Fab' fragment, 1∶100, Nanoprobes Inc., Yaphank, NY, USA) for the detection of CB1, and in a biotinylated donkey anti-guinea pig IgG (1∶200, Jackson ImmunoResearch Inc., Baltimore, Pennsylvania, USA) for the detection of vGluT1, both in 1% BSA/TBS with 0.004% saponin on a shaker for 4 hours at RT.

alBNST tissue was washed in 1% BSA/TBS and processed by a conventional avidin-biotin horseradish peroxidase complex method (ABC; Elite, Vector Laboratories Burlingame, CA, USA). Tissue was washed in 1% BSA/TBS overnight at 4°C and postfixed in 1% glutaraldehyde in TBS for 10 minutes at RT. Following washes in double-distilled water, gold particles were silver-intensified with a HQ Silver kit (Nanoprobes Inc., Yaphank, NY, USA) for about 12 minutes in the dark and then washed in 0.1 M PB (pH 7.4). Sections were preincubated subsequently with 0.05% DAB in 0.1 M PB for 5 minutes, incubated by adding 0.01% hydrogen peroxide to the same solution for 5 minutes and washed in 0.1 M PB for 2 hours at RT. Stained sections were osmicated (1% OsO_4_ in 0.1 M PB, pH 7.4, 20 minutes), dehydrated in graded alcohols to propylene oxide and plastic-embedded flat in Epon 812. 80 nm ultrathin sections were collected on mesh nickel grids, stained with uranyl acetate and lead citrate, and examined in a PHILIPS EM2008S electron microscope. Tissue preparations were photographed by using a digital camera coupled to the electron microscope.

Figure compositions were scanned at 500 dots per inch (dpi). Labeling and minor adjustments in contrast and brightness were made using Adobe Photoshop (CS, Adobe Systems, San Jose, CA, USA).

### Slice Preparation and *Ex-Vivo* Electrophysiology

Whole cell patch-clamp and extracellular field recordings were made from visualized principal pyramidal cells (projecting star neurons homologous to temporal layer V cortical pyramidal neurons [13]) in coronal slices of rat alBNST (Sprague-Dawley 250 g), as previously described [25]. In brief, rats were anesthetized with halothane and decapitated. The brain was sliced (300 µm) in the coronal plane using a vibratome (Integraslice, Campden Instruments, UK) and maintained in physiological saline at 4°C. Slices containing the alBNST were stored at 32–35°C before being placed in the recording chamber and superfused (2 ml/min) with artificial cerebrospinal fluid (ACSF) that contained (in mM): 126 NaCl, 2.5 KCl, 1.2 MgCl_2_, 2.4 CaCl_2_, 18 NaHCO_3_, 1.2 NaH_2_PO_4_, and 11 Glucose, and was equilibrated with 95% O_2_/5% CO_2_. All experiments were done at 32–35°C. Isolated excitatory postsynaptic currents (EPSCs) or inhibitory postsynaptic currents (IPSCs) were evoked pharmacologically by local stimulation within the alBNST and superfusion of picrotoxin (PTX, 100 µM) to block GABA_A_ receptors or the glutamate receptor blockers 6,7 dinitroquinoxaline-2,3-dione (DNQX, 20 µM) and DL-2-amino-5-phosphonovalerate (APV, 50 µM) to isolate GABAergic IPSCs. All drugs were added at the final concentration to the superfusion medium.

To evoke synaptic currents, stimuli (100–150 µsec duration) were delivered at 0.1 Hz through a glass electrode filled with ACSF and placed at the border between the internal capsule and the alBNST [19], [25]. Both the EPSC/IPSC area and amplitude were measured (graphs depict amplitudes).

For extracellular field recordings, the pipette was filled with ACSF. Both the field excitatory postsynaptic potential (fEPSP) area and amplitude were measured (graphs depict amplitudes). The glutamatergic nature of the extracellular fEPSP was confirmed at the end of the experiments through the application of the non-NMDA ionotropic glutamate receptor antagonist DNQX (20 µM), which completely blocked the synaptic N2 component without altering the non-synaptic N1 component.

For whole cell patch-clamp experiments, alBNST principal neurons were visualized using an upright microscope with infrared illumination. Recordings were made with whole cell electrodes containing the following (mM): cesium methane-sulfonate (CH_3_O_3_SCs) 128, NaCl 20, MgCl_2_1, EGTA 1, CaCl_2_ 0.3, Na^2+^-ATP 2, Na^+^-GTP 0.3, Glucose 10 buffered with Hepes 10, pH 7.3, osmolarity 290 mOsm. Electrode resistance was 4–6 MOhms.

A −2 mV hyperpolarizing pulse was applied before each EPSC/IPSC in order to evaluate the access resistance, and those experiments in which this parameter changed >20% were rejected. Access resistance compensation was not ordinarily used and acceptable access resistance was <25 MOhms. The potential reference of the amplifier was adjusted to zero prior to breaking into the cell. An Axopatch-1D (Axon Instruments, USA) was used to record the data, which were filtered at 1–2 kHz, digitized at 5 kHz on a DigiData 1200 interface (Axon Instruments, USA) and collected on a PC using Clampex 9.2 (Axon Instruments, USA) and analyzed using Clampfit 9.2 (Axon Instruments, USA).

Spontaneous excitatory postsynaptic currents (sEPSCs) were recorded in the whole cell voltage-clamp configuration using Axoscope 9.2 (Axon Instruments, USA). sEPSC amplitude and inter-interval time were detected and analyzed using Clampfit 9.2 (Axon Instruments, USA). For this analysis, a template of sEPSCs generated from averaging several typical synaptic events was slided along the data trace one point at a time. At each position, this template is optimally scaled and offset to fit the data and a detection criterion is calculated. The detection criterion is the template-scaling factor divided by the goodness-of-fit at each position. An event is detected when this criterion exceeds a threshold and reaches a sharp maximum.

For the estimation of the paired-pulse ratio (PPR), two stimuli were delivered with a 50 msec interval at stimulus intensity about 40–60% of the one eliciting the maximal response (∼0.2 mA). The PPR of evoked excitatory and inhibitory postsynaptic currents was calculated by dividing the mean of all 30 EPSC2 or IPSC2 (2nd evoked responses) amplitudes by the mean of all 30 corresponding EPSC1 or IPSC1 (1st evoked responses).

### Data Analysis and Materials

All values are given as mean ± S.E.M. For field recording experiments and patch-clamp experiments, *n* corresponds to the number of individual cells analyzed, with at least 4 animals included in each condition. When two means were compared considering the relation between before and after drug application, statistical significance of their difference was assessed using two-tailed paired Student's t tests. Kolmogorov-Smirnov test was used for the statistical comparison of the cumulative distributions. All statistical tests were performed with Graph Pad PRISM (version 4.0; Graph Pad Software Inc. San Diego, CA, USA) and Kyplot v2.0 beta 13 (Developed by Koichi Yoshioka; Tokyo, Japan).

The fitting of concentration response curves were calculated according to y = {y_max_−y_min_/1+ (x/EC_50_)n}+ y_min_ (where y_max_ = response in the absence of agonist, y_min_ = response remaining in presence of maximal agonist concentration, x = concentration, EC_50_ = concentration of agonist producing 50% of the maximal response and n = slope) with Kaleidagraph software (Abelbeck Software, USA).

### Quantitative Analysis of CB1 in vGluT1 + and vGluT1–Synaptic Terminals

alBNST sections from 3 rats processed for the co-localization of CB1 and vGluT1 with preembedding immunocytochemistry were used for quantitative analysis. 50-µm-thick alBNST sections from each animal showing good and reproducible DAB immunoreaction and silver-intensified gold particles were cut at 80 nm. Image-J (version 1.36) was used to measure the membrane length. Electron micrographs (10,000–25,000X) were taken from grids (132 µm side) containing DAB immunodeposits; all of them showed a similar DAB labeling intensity indicating that selected areas were at the same depth. Furthermore, to avoid false negatives, only ultrahin sections in the first 1.5 µm from the surface of the tissue block were examined. Positive labeling was considered if at least one immunoparticle was within approximately 30 nm from the plasmalemma. Metal particles on membranes and positive immunoreactive profiles were visualized and counted. Density of immunoparticles were averaged from different samples and presented as mean ± S.E.M. Group differences were compared by unpaired Student's t test (two-sided); p<0.05. The level of background labeling (0.08 particles/µm) was subtracted from the density of immunoparticles in synaptic terminals.

Percentages of vGluT1 + and vGluT1–synaptic terminals with CB1 were analyzed and displayed using a statistical software package (GraphPad Prism 4, GraphPad Software Inc, San Diego, USA).

### Drugs

Picrotoxin, and CP55,940 from SIGMA (St. Quentin Fallavier, France), DNQX, AM251, APV and WIN55,212,2 from Tocris (Bristol, UK). SR141716A was a generous gift from Sanofi-Recherche (Montpellier, France). Other chemicals were from the highest commercial grade available.
